# An Image Encryption Algorithm Based on Discrete-Time Alternating Quantum Walk and Advanced Encryption Standard

**DOI:** 10.3390/e24050608

**Published:** 2022-04-27

**Authors:** Guangzhe Liu, Wei Li, Xingkui Fan, Zhuang Li, Yuxuan Wang, Hongyang Ma

**Affiliations:** 1 School of Science, Qingdao University of Technology, Qingdao 266520, China; 202021060804@qut.edu.cn (G.L.); hdshx003@qut.edu.cn (X.F.); 201911060153@qut.edu.cn (Z.L.); 2 School of Information and Control Engineering, Qingdao University of Technology, Qingdao 266520, China; 202021050678@qut.edu.cn; 3 School of Electronic and Information Engineering, Soochow University, Suzhou 215000, China; 1929401105@stu.suda.edu.cn

**Keywords:** image encryption, discrete-time alternating quantum walk, advanced encryption standard, chaotic scramble, keystream generator

## Abstract

This paper proposes an image encryption scheme based on a discrete-time alternating quantum walk (AQW) and the advanced encryption standard (AES). We use quantum properties to improve the AES algorithm, which uses a keystream generator related to AQW parameters to generate a probability distribution matrix. Some singular values of the matrix are extracted as the key to the AES algorithm. The Rcon of the AES algorithm is replaced with the elements of the probability distribution matrix. Then, the ascending order of the size of the clone probability distribution matrix scrambles the mapping rules of the S-box and ShiftRow transformations in the AES algorithm. The algorithm uses a probability distribution matrix and plaintext XOR operation to complete the preprocessing and uses the modified AES algorithm to complete the encryption process. The technology is based on simulation verification, including pixel correlation, histograms, differential attacks, noise attacks, information entropy, key sensitivity, and space. The results demonstrate a remarkable encryption effect. Compared with other improved AES algorithms, this algorithm has the advantages of the original AES algorithm and improves the ability to resist correlation attacks.

## 1. Introduction

In the current era of big data, data protection has received more and more attention, of which image data account for a large part. Accordingly, image data protection has become the focus of the current research. With the passage of time, fruitful research results have been achieved in the field of image processing [1,2,3,4,5,6,7]. In 1999, the United States established the data encryption standard (DES) as the data processing standard [8]. This was followed by the emergence of the Rijndael algorithm, whose performance was better than the DES algorithm. On 2 October 2000, the Rijndael algorithm of block ciphers replaced the DES and was defined as the advanced encryption standard (AES). However, traditional encryption schemes such as the AES and DES are suitable for text data encryption, but not for image encryption [9,10,11]. Inspired by the AES algorithm, several researchers have improved the AES algorithm in order for it to better adapt to the encryption of image data. In recent years, the improved AES algorithm has still been favored by researchers. In 2017, Xu et al. [12] proposed an improvement of the S-box of the AES algorithm based on FPGA. They designed a secure processor model of a chaotic neural network, realized the chaotic characteristics of the S-box, and improved the ability of the anti-attack system. In 2019, Arab et al. [13] proposed an image encryption method based on a chaotic system and the AES algorithm. The Arnold chaotic sequence provides the encryption key for the improved AES algorithm. This method not only reduces the time complexity of the algorithm, but also increases the difference fusion ability of the algorithm, so that the image encrypted by the algorithm can resist the difference attack. In 2021, Lin et al. [14] proposed a dynamic random key improved AES cryptosystem based on chaotic synchronization. It can eliminate the shortcomings of key storage and enhance the security of encryption.

As people became aware of the AES algorithm, the risk of cracking the algorithm also increased. When attackers intercept a round of extended keys and encrypted plaintext, there may be the flaw of plaintext disclosure [15,16,17,18,19]. The traditional AES encryption algorithm may be cracked because of its fixed initial key and the invariance of the S-box [20]. The reverse process is the main approach adopted by the key breaker. If we can find a way to change the algorithm process, we can effectively prevent attackers from attacking the plaintext. To improve this defect, it has been proven that the quantum walk (QW) has the characteristics of chaotic dynamics and can be applied to cryptography [21]. Many scholars combined the quantum random walk with image encryption to acquire higher security. For example, in 2019, Abd EL-Latif et al. [21] proposed a pseudo-random number generator (PRNG) using a controlled alternating quantum walk. The quantum color image is encrypted by a quantum controlled NOT (C-NOT) gate controlled by the PRNG. It has good pseudo-random characteristics and vital qualities for viable applications. In 2021, Abualigah et al. [22] proposed a new meta-heuristic method called the arithmetic optimization algorithm (AOA). Compared with other well-known optimization algorithms, this algorithm is effective at solving challenging optimization problems. Then, they proposed a population-based optimization method called Aquila Optimizer (AO) [23], which was inspired by Aquilas’ behaviors in nature during the process of catching prey. Compared with existing meta-heuristic algorithms, this algorithm demonstrates certain advantages. In 2021, Bassem Abd-El-Atty et al. [24] used the AQW to generate two random masks for double random phase encoding, making the color image more secure. Numerous research results emphasize the importance of the chaotic characteristics, unpredictability and aperiodicity of quantum technology in image encryption.

This study proposes a new encryption scheme that combines quantum technology with the advanced encryption standard (AES) algorithm. In this scheme, the alternating quantum walk in discrete time is used as the keystream generator, which can provide a probability distribution matrix with chaotic dynamics. Theoretically, the probability distribution matrix cannot be deciphered without obtaining the quantum walking parameters. The main contributions of this study are summarized as follows:

(1) The algorithm extracts the singular values of the matrix as keys to the improved AES algorithm. In terms of key security, quantum technology provides a theoretically secure key to the AES algorithm, effectively preventing an attacker from intercepting the key.
(2) The element of the matrix replaces the Rcon, and the Rcon defined by the AES algorithm is replaced by a fixed value for a variable related to the alternating quantum walk parameters. The Rcon is closely related to the key expansion function of the AES algorithm, and the size of the key group elements varies with changes in the Rcon to overcome the shortcomings of the fixed key of the AES algorithm and enhance the randomness of the key group of the AES algorithm.
(3) Because of the uncertainty of the size of the matrix elements, the ascending order of the size of the clone probability distribution matrix scrambles the mapping rules of the S-box and ShiftRow transformations in the AES algorithm. It combines the chaotic dynamics of the matrix with the AES algorithm, enhancing the random and scrambling performances of the AES algorithm.

The remainder of this paper is organized as follows. In Section 2, we introduce the principle of the AQW and modified AES algorithm and provide a keystream generator based on the quantum random walk. The proposed principle of encryption and decryption is presented in Section 3. The experimental simulation and performance analysis of our algorithm are in Section 4. Finally, the summary and prospect are drawn in Section 5.

## 2. Algorithm Principle

### 2.1. Alternate Quantum Walks

**Figure 1:**
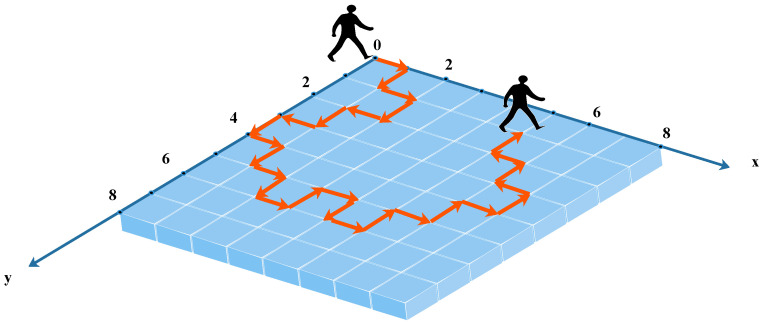
The 2D alternating quantum walks. Movement rule of the walker: the walker can choose to walk in the upper, lower, left, and right directions; however, the walker can only choose one direction at a time, and the distance of each walk is 1.

The position probability distribution of quantum walkers is different from that of classical walkers, which gives quantum walkers more unique properties, which is conducive to the proposal of quantum algorithms and has been widely used in the development of quantum algorithms [25,26,27,28,29,30]. For example, the most intuitive 1D discrete-time quantum walk, which has quantum parallelism, is known as online walking. Because of its simple form, there are many research developments at present [31,32,33,34,35,36,37,38,39]. The 2D alternating quantum walk acts on the Cartesian coordinate system, and the walking range of the walker is controlled by the initial parameters. The approximate process of the two-dimensional alternating quantum walk is shown in Figure 1.

The Hilbert space of the whole quantum walking system can be expressed by the direct product of the walker’s position space and the coin state space: ${\hat{H}}_{w}⊗{\hat{H}}_{c}$ [40], a quantum as a walker, where the walker’s position space ${\hat{H}}_{w}$ is composed of position vectors |x,y〉(x,y∈Z) and the coin space ${\hat{H}}_{c}$ consists of the linear combination of two basic vectors |c〉(c=0,1) of the coin state, ${\hat{H}}_{c}=cos\alpha |0\rangle +sin\alpha |1\rangle$.

The coin operator $\hat{C}$ is a function of θ, where $\alpha ,\theta \in [0,\frac{\pi }{2}]$. The AQW controls the walker by selecting the initial parameters (N,T,α,θ). The walker walks in a 2D Cartesian coordinate system. In short, the walker walks first along the *X* axis, then once along the *Y* axis, alternately, where *N* × *N* is the range of walking and *T* is the number of steps. Before each step, the walker acts the coin operator $\hat{C}$ on the coin state and moves the position according to the state of the coin and the shift operator $\hat{S}$. The specific process can be represented by the transformation operator $\hat{U}$, which can be formulated as Equation (1):

(1) $$\hat{U}=\sum_{y}{\hat{S}}_{y}\left(I⊗\hat{C}\right)\sum_{x}{\hat{S}}_{x}\left(I⊗\hat{C}\right)$$

where ${\hat{S}}_{y}$ and ${\hat{S}}_{x}$ are shift operators defined in the Y and X axes, respectively. I is the unit operator. The shift operator ${\hat{S}}_{y}$ is similar to ${\hat{S}}_{x}$, and ${\hat{S}}_{y}$ is shown in Equation (2):

(2) $$\begin{array}{cc} {\hat{S}}_{y}= & \sum_{x,y}^{N}\left(|x,\left(y+1\right)mod\;N,0\rangle \langle x,y,0\right|\\ \; & +|x,(y-1)mod\;N,0\rangle \langle x,y,1|) \end{array}$$

The coin operator $\hat{C}$ is a function of θ.

(3) $$\hat{C}=\left(\begin{array}{cc} cos\theta & sin\theta \\ sin\theta & -cos\theta \end{array}\right)$$

After the walker walks *T* steps, the evolution relationship between the state of the system ${|\psi \rangle }_{T}$ and the initial state of the system ${|\psi \rangle }_{0}$ can be calculated by Equation (4):

(4) $${|\psi \rangle }_{T}={\left(\hat{U}\right)}^{T}{|\psi \rangle }_{0}$$

Through quantum measurement, the probability of detecting a walker at the position (x,y) can be expressed by Equation (5):

(5) $$P\left(x,y,T\right)=|\langle x,y,0|{\left(\hat{U}\right)}^{T}{|\psi \rangle }_{0}{|}^{2}+|\langle x,y,1|{\left(\hat{U}\right)}^{T}{|\psi \rangle }_{0}{|}^{2}$$

After measuring the probability of quantum occurrence, the AQW can generate a probability distribution matrix of *N* × *N*. The AQW is used as a keystream generator and matrix elements as a special key stream. The size and arrangement of the elements in the matrix have the characteristic of chaos. On the one hand, the singular value of the matrix is extracted to provide a security key for the algorithm of Section 2.2. On the other hand, the submatrix of the probability distribution matrix is randomly extracted for the quantum scrambling of the traditional AES algorithm.

### 2.2. Modified AES Algorithm

In general, the original AES algorithm first groups the plaintext into several groups of blocks with a size of 4 × 4 and then encrypts each block. The plaintext grouping in the AES algorithm is described by a square matrix with bytes as units, which is called a state matrix. This is because the pixel value range of the image data is in [0, 255], and the pixel value can be represented by an 8 bit binary number, which is exactly 1 byte.

**Figure 2:**
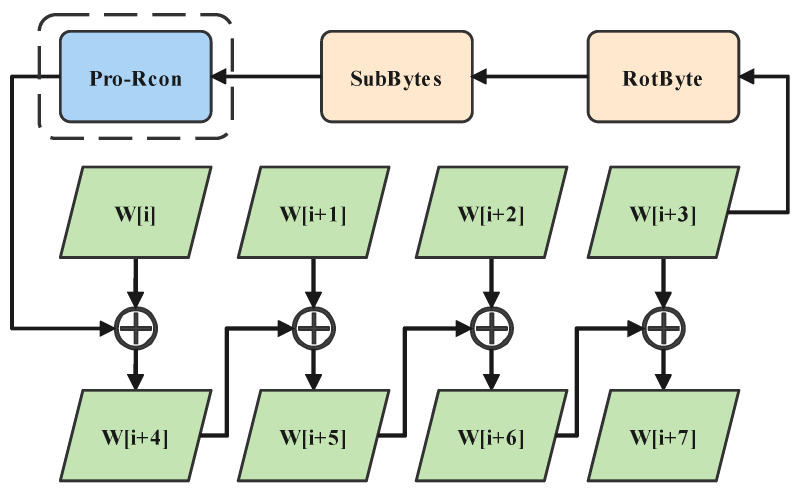
Key expansion. Pro-Rcon is in the blue box, and key group W[i] is in the green box. When the input of the key extension function is W[0]∼W[3], the key extension function can obtain the extended key W[4]∼W[43].

**Figure 3:**
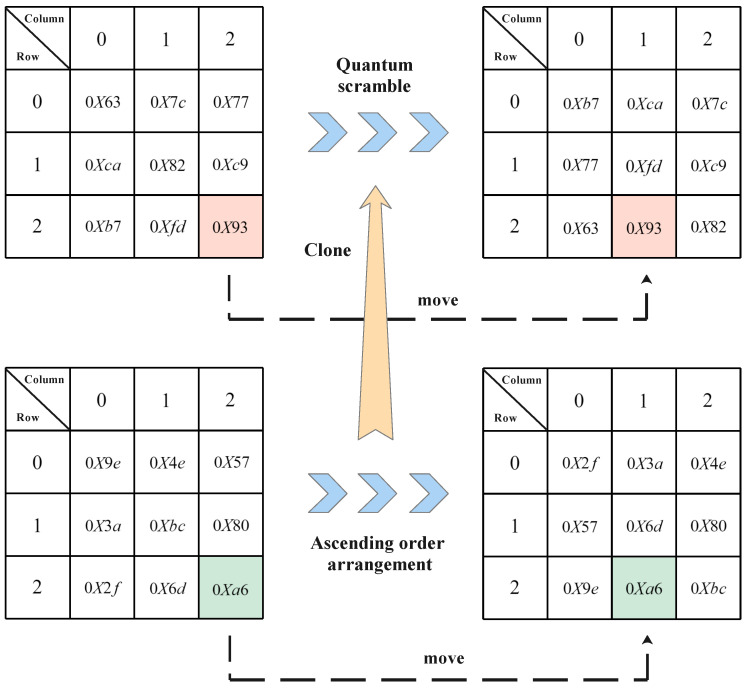
Pro-ByteSub transformation. The elements of the probability distribution matrix are in the green box, and the elements in the S-box are in the red box. The elements of S-box are scrambled by the ascending order of the elements of the clone probability distribution matrix.

**Figure 4:**
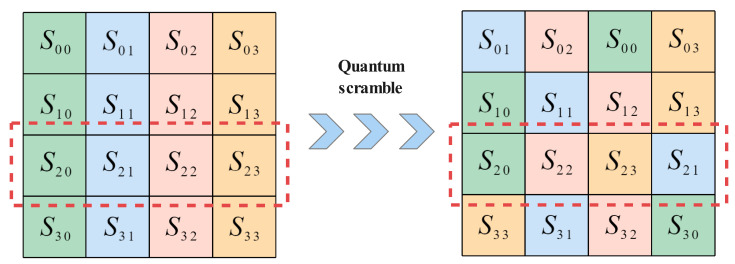
Pro-ShiftRow transformation.

A quantum walk can resist the possible attacks of digital computers and quantum computers, and it is a good tool for designing modern cryptographic mechanisms. The traditional AES encryption algorithm has the risk of cracking plaintext because of its fixed key expansion function and encryption process. This paper puts forward three new concepts: Pro-Rcon, Pro-ByteSub, and Pro-ShiftRow. Their improvement of the AES algorithm is as follows:

1. Pro-Rcon: We change the original key expansion function, use the keystream generator to provide 10 submatrices of 4 × 1 size, and convert matrix elements to hexadecimal numbers, each containing 4 bytes. Rcon[j] is 8 bytes, where j ∈[1,10]. Rcon[j] defined by the original AES is replaced with a submatrix, and Rcon[j] is changed from the original fixed value to a variable Pro-Rcon[j] related to the initial parameters of the AQW. Pro-Rcon[j] changes the key expansion process, which in turn affects the whole encryption process. The key extension flowchart is shown in Figure 2, where i ∈[0,36].

2. Pro-ByteSub: We change the S-box mapping rules of ByteSub transformation during the original encryption process. A 16 × 16 size submatrix is provided by the keystream generator, and the elements in the submatrix are converted into hexadecimal numbers, so that the substitution table of the S-box mapping rules corresponds to the elements in the same position of the submatrix. Because of the different sizes of the elements of the submatrix, the elements in the submatrix are arranged in ascending order, and the position of each element is shifted. Then, we clone the moving order of matrix elements, to scramble the mapping of the S-box. We briefly explain the law of Pro-ByteSub transformation to a part of the S-box, as shown in Figure 3.

3. Pro-ShiftRow: We change the ShiftRow transformation in the original encryption process and use the keystream generator to provide a 4 × 4 size submatrix, which is similar to the scrambling law in Pro-ByteSub to scramble the state matrix. We convert the elements of the submatrix to hexadecimal numbers so that the state matrix corresponds to the elements in the same position as the submatrix. The state matrix and the submatrix have four elements in each row, which are arranged in ascending order according to each row of the submatrix; the moving order of each row of elements is cloned, and the state matrix is scrambled accordingly, as shown in Figure 4.

The encryption process of AES algorithm is a repetitive process. When a certain operation process changes, it will directly affect the input of the next round of encryption and then change the output of each round. It not only applies the anti-interference of the quantum walk to the AES algorithm, but also increases the flexibility of the AES algorithm. At the same time, the improved scheme is reversible, and in each round of decryption process, executing the corresponding AddRoundKey, we can obtain the decryption pixel matrix, which ensures the integrity of the decryption.

## 3. Principle of Encryption and Decryption

### 3.1. Encryption Algorithm

The key length of this scheme is 128 bits, and the number of encryption rounds is 10. We stipulate that the operations are performed sequentially: Pro-ByteSub, Pro-ShiftRow, MixColumn, and AddRoundKey, which is a round of processing. To facilitate a better description in Step 5, the specific encryption steps are as follows:

Step 1: Select the parameter (N,T,α,θ), and execute the *T*-step AQW on the loop on *N* × *N* vertices. The purpose is to obtain the probability distribution matrix *P* of *N* × *N* size and use the bicubic interpolation scaling technique to convert the matrix *P* to the same size as plaintext I(m×n).

Step 2: Using Equation (6), convert the elements in the matrix *P* to the integer values in [0, 255]. The matrix $\hat{P}$ and plaintext perform the bitwise XOR operation to complete the preprocessing of plaintext.

(6) $$\hat{P}=fix\;\left(\bar{P}*10^{12}\right)\;mod\;256$$

Step 3: The singular value of the matrix $\hat{P}$ is converted into the corresponding hexadecimal number, and the first 32 bits of the singular value are used as the key of the improved AES algorithm. Here, the 32 bits in hexadecimals are 128 bits in binary.

**Figure 5:**
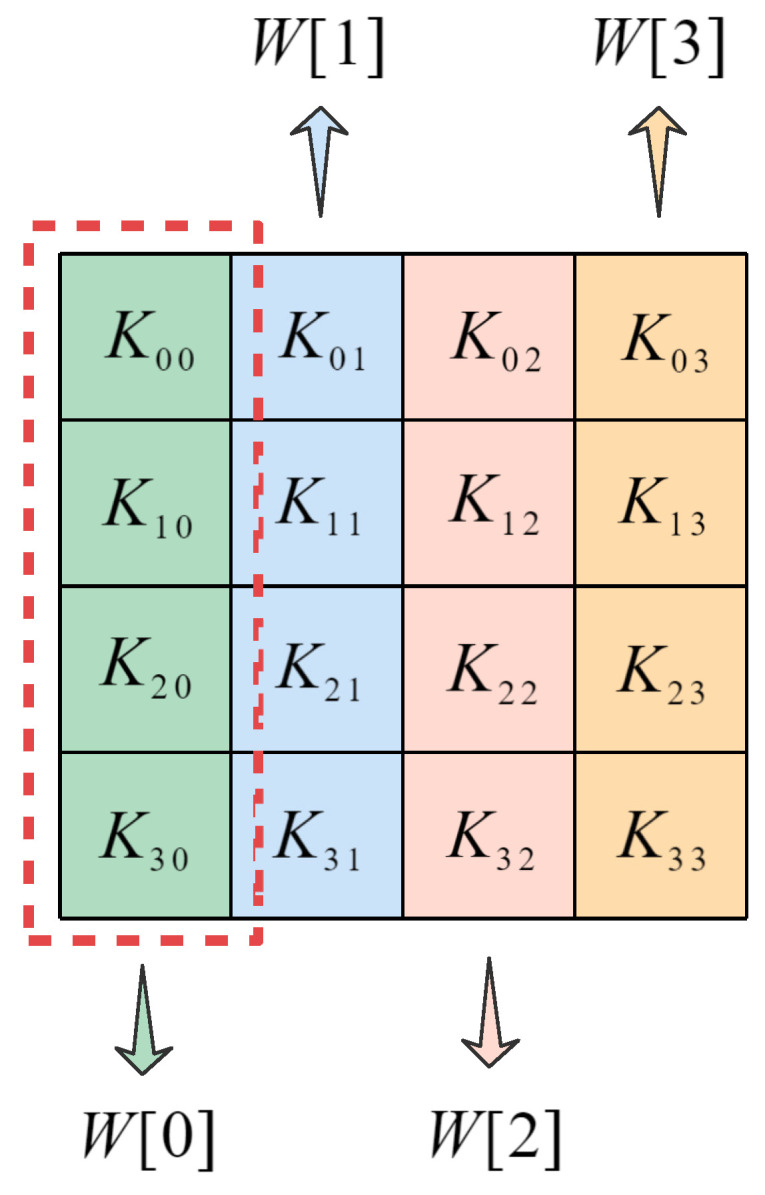
Plaintext and key grouping.

Step 4: Round Key includes *W*[0]∼*W*[43]. Taking *W*[0]∼*W*[3] as the input of the improved key extension function, the remaining extended key *W*[5]∼*W*[43] is obtained. Complete the key expansion process, as shown in Figure 5.

Step 5: We repeat nine rounds of processing in accordance with the prescribed process of one round of processing. However, the 10th round is different from the first nine rounds, performing operations in order: Pro-ByteSub, Pro-ShiftRow, and AddRoundKey.

**Figure 6:**
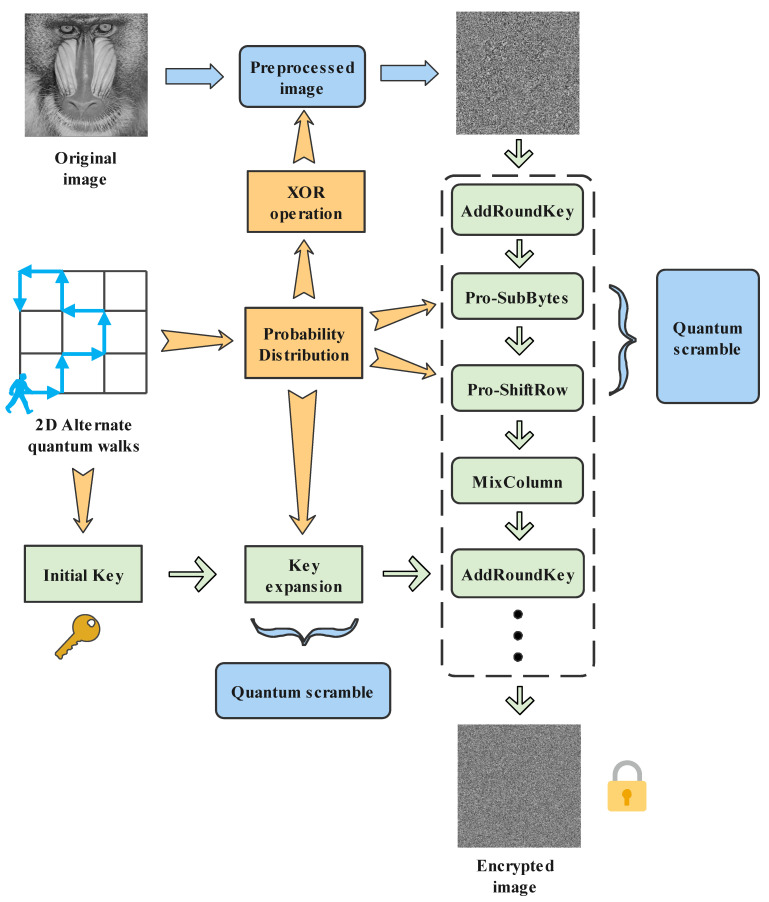
Encryption process. The quantum scrambling of the image preprocessing and encryption process by quantum technology are in the blue box, and the general process of improving the AES algorithm is in the green box.

Step 6: After the plaintext is encrypted twice, all the encryption processes are complete. The encryption process is shown in Figure 6.

### 3.2. Decryption Algorithm

Decryption is the reverse of encryption. We stipulate that the operations are performed sequentially: Inv Pro-ByteSub, Inv Pro-ShiftRow, Inv MixColumn, and AddRoundKey, which is a round of processing. To facilitate a better description in Step 7, the specific decryption steps are as follows:

Step 7: Perform the AddRoundKey operation first. Then, we repeat nine rounds of processing in accordance with the prescribed process of one round of processing. Similarly, the 10th round is different from the first nine rounds, performing the operation in order: Inv Pro-ByteSub, Inv Pro-ShiftRow, and AddRoundKey.

**Figure 7:**
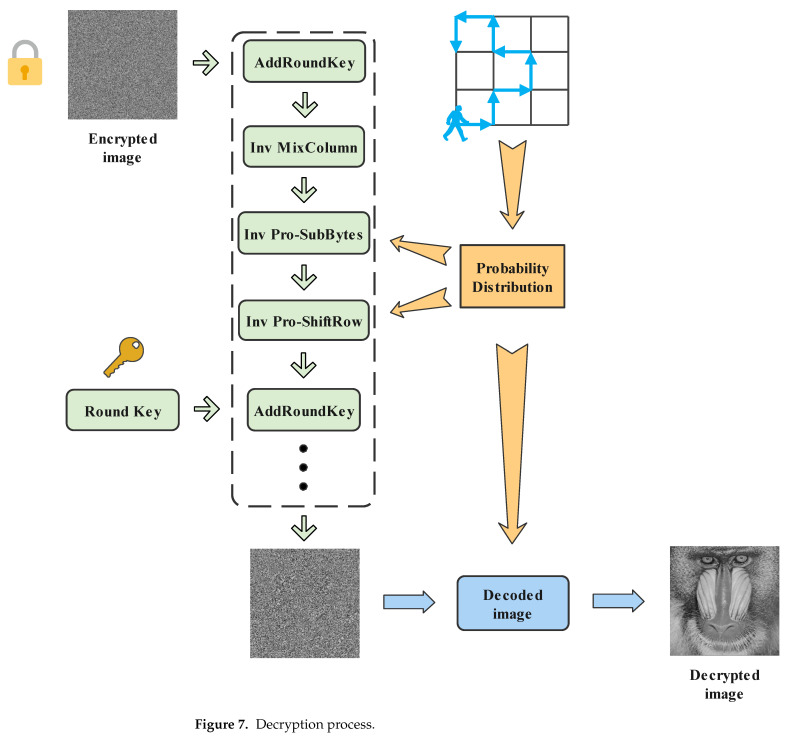
Decryption process.

Step 8: Perform the bitwise XOR operation with matrix $\hat{P}$ to complete the whole decryption process. The decryption process is shown in Figure 7.

## 4. Experimental Simulation and Performance Analysis

According to the proposed scheme, the security of the encryption algorithm was analyzed, and a series of experiments was performed using images of baboon, pepper, and house, each of size 512 × 512 pixels. In addition, the initial parameter of the AQW was set to $\left(N=500,T=501,\alpha =\frac{\pi }{4},\theta =\frac{\pi }{3}\right)$. The performance of the algorithm was evaluated by standard metrics and tests such as pixel correlation, histogram, differential attack, noise attack, information entropy, key sensitivity, and space.

### 4.1. Encryption Effect

**Figure 8:**
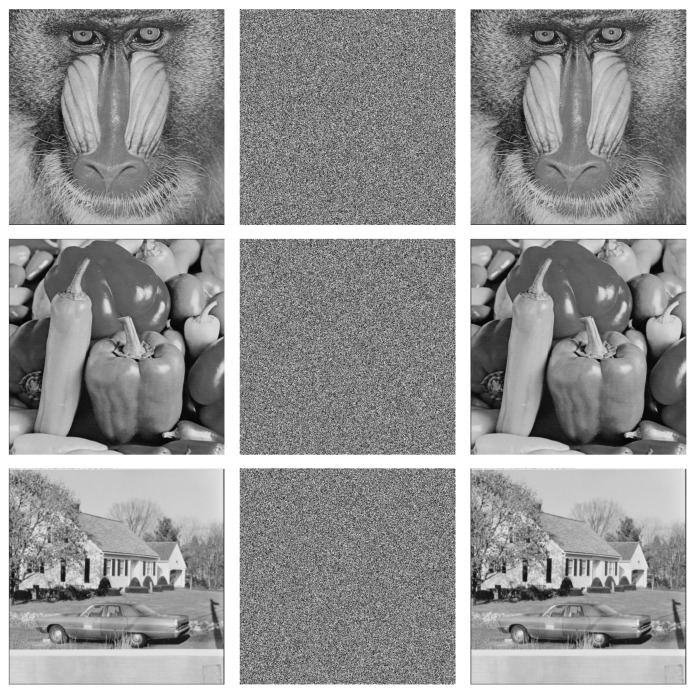
Encryption result analysis. The first column: plaintext images; the second column: encrypted images; the third column: decrypted images.

Under a Windows system, the simulation experiment was executed with Python 3.7. After the plaintext had been encrypted and decrypted, we could hardly see any information of the plaintext image, which plays the role of information confidentiality. The plaintext image was compared with the decrypted image, and the decrypted image was exactly the same as the original image. The encrypted and decrypted images of baboon, peppers, and house are shown in Figure 8.

### 4.2. Histogram Analysis

**Figure 9:**
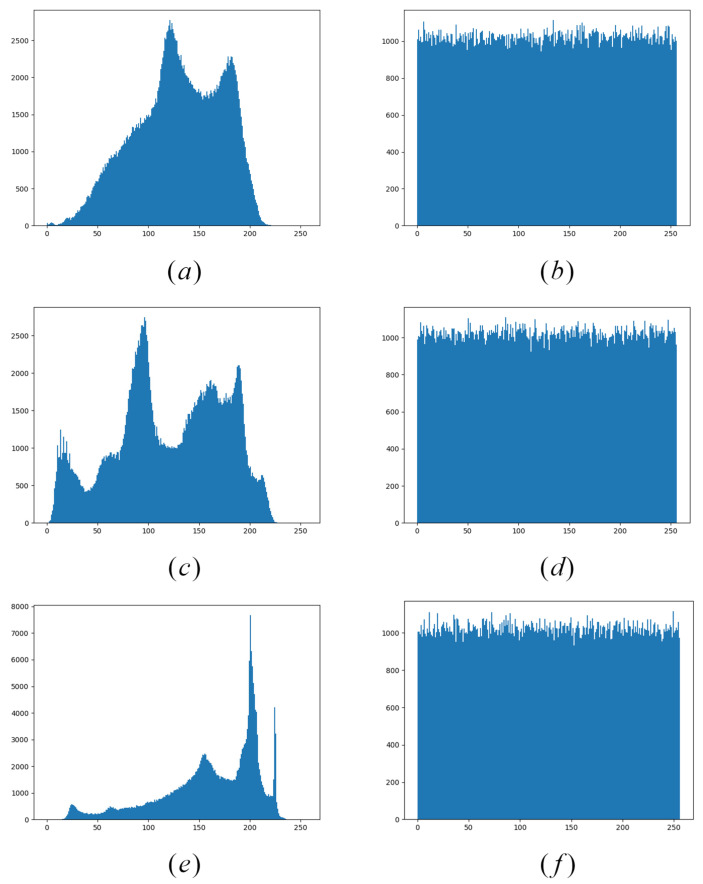
Histogram analysis of plaintext and ciphertext. Plaintext: (**a**) baboon, (**c**) peppers, (**e**) house; ciphertext: (**b**) baboon, (**d**) peppers, (**f**) house.

From the point of view of the plaintext and ciphertext histogram, the distribution of pixel values tends to be consistent. The algorithm balances the frequency of each pixel value, retains the ability of the original AES algorithm to resist statistical attacks, and hides the information of image pixels well. The histogram of the plaintext and corresponding ciphertext is shown in Figure 9.

### 4.3. Correlation Analysis

In the horizontal, vertical, and diagonal directions of adjacent pixels in the plaintext and ciphertext, 3000 pairs of adjacent pixels are randomly selected to test the correlation, and the correlation coefficient $C_{AB}$ of each group of data is calculated. $C_{AB}$ is calculated by Equation (7).

(7) $$C_{AB}=\frac{\sum_{n=1}^{N}\left(A_{n}-\bar{A}\right)\left(B_{n}-\bar{B}\right)}{\sqrt{\sum_{n=1}^{N}{\left(A_{n}-\bar{A}\right)}^{2}{\left(B_{n}-\bar{B}\right)}^{2}}}$$

where $A_{n}$ and $B_{n}$ represent the values of adjacent pixels, $\bar{A}$ and $\bar{B}$ represent the average value of adjacent pixels, and *N* represents the total number of pairs of adjacent pixels.

**Figure 10:**
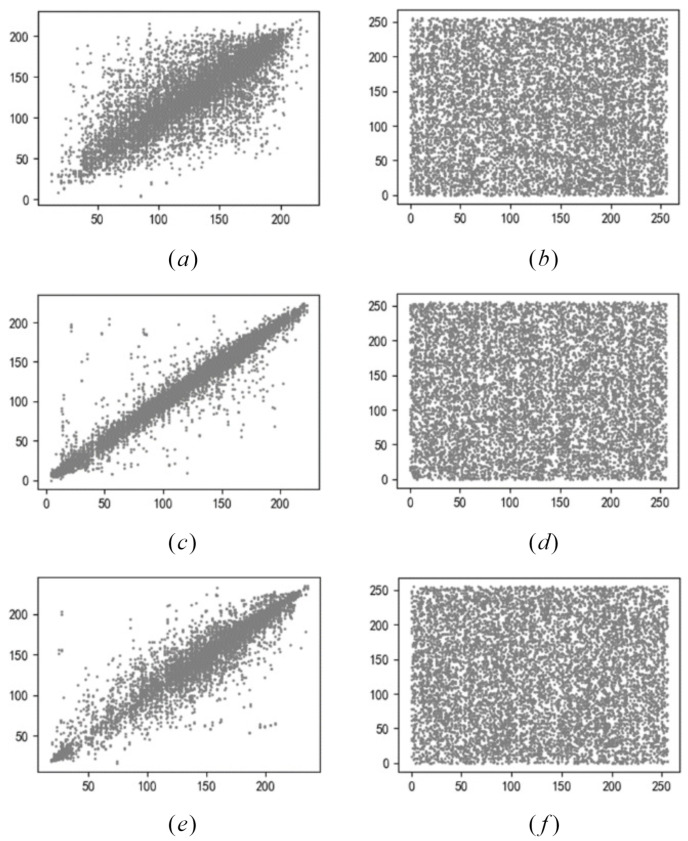
Correlation analysis of plaintext and ciphertext. Plaintext: (**a**) baboon, (**c**) peppers, (**e**) house; ciphertext: (**b**) baboon, (**d**) peppers, (**f**) house.

As summarized in Table 1, the correlation coefficients between ciphertext pixels vary greatly, and the coefficients are close to 0; the correlation between pixels disappears, and the encryption effect is remarkable. Based on the original AES algorithm, we combined quantum technology with the AES algorithm and used an alternating quantum walk (AQW) to generate a matrix with quantum properties. In particular, the matrix has certain chaotic characteristics: unpredictable and aperiodic. The scheme uses these characteristics of the matrix to scramble the Rcon, ByteSub transformations, and ShiftRow transformations of the AES algorithm, which increases the random and scrambling performances of each step in the encryption iteration of the AES algorithm. This enhances the complexity of the algorithm. Accordingly, the ability of the scheme to resist correlation attacks is significantly improved. The correlation coefficients of plaintext and ciphertext pixels in three directions were analyzed, as shown in Figure 10.

### 4.4. Comparison with Other Image Encryption Schemes

The algorithm averages the pixel correlation coefficients of the baboon, peppers, and house encrypted images in the horizontal, vertical, and diagonal directions, respectively. The average in the horizontal direction is 0.0030 ((0.0026 + 0.0045 + 0.0020)/3). The average in the vertical direction is 0.0033 ((0.0043 + 0.0042 + 0.0016)/3). The average in the diagonal direction is 0.0030 ((0.0034 + 0.0018 + 0.0016)/3). In addition, we also analyzed the information entropy and took the average of the information entropy of the encrypted images of baboon, peppers, and house; the average is 7.999 ((7.999 + 7.999 + 7.999)/3). The average values of the pixel correlation coefficient in the three directions and information entropy were compared with other schemes [41,42,43]. The ciphertext pixel correlation coefficient of the proposed algorithm was more stable and close to 0. The ciphertext information entropy of the proposed algorithm was close to 8. The results demonstrated that the information random performance of the ciphertext was better, and it was better than other algorithms in correlation analysis and information entropy, as summarized in Table 2.

### 4.5. Analysis of Differential Attack

Differential attack is a selective plaintext attack, where the attacker makes minor changes to the plaintext image, uses the encryption algorithm to encrypt the image before and after the change, and compares two ciphertext images to crack the ciphertext and find the relationship between the two ciphertext images. The performance of the resistance to differential attacks depends on the sensitivity to plaintext. In the field of image encryption, there are two very important variables to measure the difference between two images: the number of pixel change rate (NPCR) and unified average change intensity (UACI).

To resist the differential attack, when the pixel of the plaintext image changes, the greater the ciphertext image change, the stronger the ability to resist the differential attack is. Ideally, the NPCR value is 99.6094% and the UACI value is 33.4635%. The NPCR and UACI can be calculated by

(8) $$\left\{\begin{array}{c} NPCR=\frac{\sum_{i,j}D\left(i,j\right)}{M*N}*100\%\\ UACI=\frac{1}{M*N}\frac{\sum_{}\left(C_{1}\left(i,j\right)-C_{2}\left(i,j\right)\right)}{255}*100\% \end{array}\right.$$

where *M* and *N* are the width and height of the two images; D(i,j) is defined as

(9) $$D\left(i,j\right)=\left\{\begin{array}{c} 1,C_{1}\left(i,j\right)\neq C_{2}\left(i,j\right)\\ 0,otherwise \end{array}\right.$$

$C_{1}\left(i,j\right)$ and $C_{2}\left(i,j\right)$ represent the pixel values of the two ciphertexts at point (i,j).

We changed the pixel value of the plaintext (0,0) point to 0, and the other pixel values remained the same. Then, we compared the NPCR and UACI values of the ciphertext before and after the change, as summarized in Table 3.

The experimental data show that the NPCR value and UACI value of our scheme were close to the ideal value. The modified AES algorithm was more sensitive to the change of the plaintext than the original AES algorithm, and the modified AES algorithm had strong anti-differential attack ability and high security performance.

### 4.6. Noise Analysis

**Figure 11:**
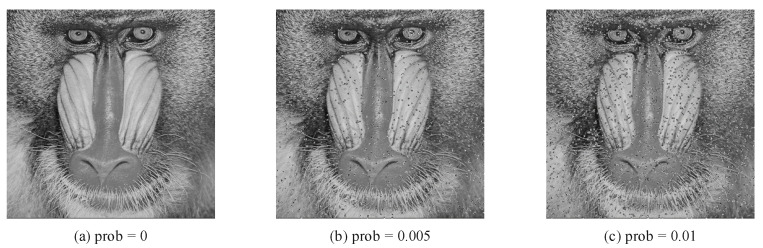
(**a**) shows a decrypted image that is not affected by salt and pepper noise. (**b**,**c**) show decrypted images affected by salt and pepper noise with intensities of 0.005 and 0.01.

In the practical world, the transmission of images on the Internet will inevitably be affected by various factors, such as noise. Distortion, degradation, and pollution caused by communication noise are very common. These factors will have a certain impact on the decryption of the ciphertext. It is very difficult to recover an image from a noisy ciphertext. Therefore, the anti-noise ability is an important index to test the performance of the encryption scheme. The image encryption algorithm must be robust enough to resist noise attacks in practical scenes. Salt and pepper noise is caused by the signal pulse intensity. In this method, different degrees of salt and pepper noise are added to the ciphertext for simulation experiments. The ciphertext was decrypted with salt and pepper noise of 1% and 0.5%, respectively, and the decryption results are compared as shown in Figure 11.

As shown in Figure 11, after the decrypted image is affected by different intensities of salt and pepper noise, the main information of the plaintext can still be recognized by human eyes. Thus, the proposed algorithm has a certain ability to resist noise attacks.

### 4.7. Key Space and Key Sensitivity Analysis

An efficient cryptosystem should provide enough key space. Assuming that the calculation accuracy is in the order of the magnitude and the four initial values of the AQW (N,T,α,θ) are the key parameters, the key space of the AQW system is calculated as: $10^{64}\approx 2^{210}$, and the key length of the AQW is 210 bits. In addition, because the key space of the AES algorithm is 256 bits, the key length of the algorithm can reach 466 bits. This is enough for optical image encryption [44]. In addition, quantum technology provides a theoretically secure key for the AES algorithm, which can effectively resist the interception of keys by attackers.

**Figure 12:**
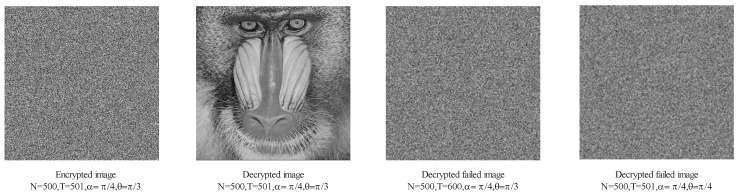
Decrypted images with different keys.

In general, if the decryption key changes, the decrypted image will change significantly, which is the key sensitivity. Through the experimental simulation, the AQW parameter and the AES key constitute the key of this scheme, in which the AES key is determined by the AQW parameter. We used the correct decryption method to decrypt the baboon ciphertext and obtain the decrypted image. We changed the AQW parameter $\left(N=500,T=501,\alpha =\frac{\pi }{4},\theta =\frac{\pi }{3}\right)$ to $\left(N=500,T=600,\alpha =\frac{\pi }{4},\theta =\frac{\pi }{4}\right)$, and the decrypted image after the parameter modification is shown in Figure 12. Unless the key is completely correct, the image cannot be decrypted successfully, which indicates that the key sensitivity is high and the avalanche effect is significant.

## 5. Conclusions

This study proposed an improved AES algorithm that uses the probability distribution matrix generated by the AQW as the keystream generator. By applying the AQW to the traditional AES algorithm, the unique chaotic dynamics of the AQW were combined with the AES algorithm, theoretically providing a secure key for the algorithm. The key changes according to the parameters of the AQW, overcoming the shortcomings of the traditional AES algorithm with a fixed key and encryption process and effectively preventing the attacker from intercepting the key. This study proposed three new concepts: Pro-Rcon, Pro-ByteSub, and Pro-ShiftRow. In the AES algorithm, the key, Pro-Rcon, Pro-ByteSub transformation, and Pro-ShiftRow transformation vary with the changes in the AQW parameters, which retain the robustness and advantages of the traditional AES algorithm and enhance the random and scrambling performances of the algorithm. Finally, histogram, correlation, differential attack, information entropy, anti-noise, key space, and key sensitivity analyses were conducted. Their results demonstrated the effectiveness of the proposed scheme. Quantum technology has good application prospects in image security. However, note that with an increasing amount of information in the image data, the larger the image size, the longer the runtime of the algorithm. In the future, we will continue to study how to effectively improve the algorithm efficiency and reduce its runtime. We will study various images, such as color, medical, and remote sensing images, to improve the applicability and practicability of the algorithm.

## Figures and Tables

**Table 1:** Pixel correlation coefficients of plaintext and ciphertext.

Image	Original			Encrypted		
	Horizontal	Vertical	Diagonal	Horizontal	Vertical	Diagonal
Baboon	0.8710	0.7767	0.7530	0.0026	−0.0043	0.0034
Peppers	0.9836	0.9837	0.9717	0.0045	−0.0042	−0.0018
House	0.9529	0.9602	0.9268	0.0020	−0.0016	−0.0016

**Table 2:** Performance comparison between our scheme and other schemes.

Encryption Scheme	Horizontal	Vertical	Diagonal	Entropy
Classic AES	0.0117	−0.0273	0.0173	7.998
Our scheme	0.0030	0.0033	0.0022	7.999
Modified AES [41]	−0.0112	−0.0813	0.0009	7.999
Modified AES [42]	−0.0410	−0.0378	−0.0548	7.998
Modified AES [43]	−0.0085	−0.0081	−0.0024	7.998

**Table 3:** Analysis of resistance to differential attack.

Encryption Scheme	Image	NPCR	UACI
Classic AES	Baboon	99.5913	33.4527
	Peppers	99.5975	33.4754
	House	99.6124	33.4721
Our scheme	Baboon	99.5934	33.4551
	Peppers	99.6052	33.4717
	House	99.6101	33.4610

## Data Availability

The data are contained within the article.
